# Omni-directional wind-driven triboelectric nanogenerator with cross-shaped dielectric film

**DOI:** 10.1186/s40580-021-00276-5

**Published:** 2021-09-02

**Authors:** Yoseop Shin, Sungjun Cho, Sejin Han, Gun Young Jung

**Affiliations:** School of Materials Science and Engineering, Gwangju Institute of Science and Technology (GIST), 123 Cheomdan-gwagiro, Buk-gu, Gwangju, 61005 Republic of Korea

**Keywords:** Energy harvesting, Triboelectric nanogenerator, Wind energy, Electrification, Electrostatic induction

## Abstract

Triboelectric nanogenerators (TENGs) are actively being researched and developed to become a new external power unit for various electronics and applications. Wind is proposed as a mechanical energy source to flutter the dielectric film in wind-driven TENGs as it is clean, abundant, ubiquitous, and sustainable. Herein, we propose a TENG structure with dielectric films bent in four directions to collect the wind energy supply from all directions, unlike the conventional wind-driven TENGs which can only harvest the wind energy from one direction. Aluminum (Al) layer was intercalated within the dielectric film to improve electrostatic induction, resulting in improved triboelectric performances. Maximum open-circuit voltage (V_oc_) of 233 V, short-circuit current (I_sc_) of 348 µA, and output power density of 46.1 W m^− 2^ at an external load of 1 MΩ under a wind speed of 9 m s^− 1^ were revealed, and it faithfully lit “LED” characters composed of 25 LEDs.

**Supplementary Information:**

The online version contains supplementary material available at 10.1186/s40580-021-00276-5.

## Introduction

With the advent of the fourth industrial revolution, the Internet of Things (IoT) technology that allows data to be sent and received in real time by attaching sensors to objects is developing rapidly [1–3]. Wearable devices that are directly related to IoT are also being rapidly commercialized [4–6] and are attached close to the body of users in the forms of glasses [7], wristwatches [8], and shoes [9] for data collection from the surroundings. Therefore, the wearables must be small, light, and wireless [10]. Lithium-ion batteries are normally used as an external power source to operate these sensors and portable devices [11], but have the disadvantages of recharging requirement, replacement due to the limited lifetime and power capacity, and the risk of explosion [10, 12]. Therefore, development of new power supplies that can overcome the limitations of lithium-ion batteries is essential.

Currently, a variety of wireless sensors using IoT are ubiquitously utilized in military [13], industrial [14], academic [15], and leisure [16] fields, rendering a growing interest in permanent energy harvesting, in which the wasted and natural energies can be collected and utilized from the surrounding environment. Energy harvesting technologies convert heat [17], light [18], and mechanical energy [19] into electrical energy. Today, solar cells are one of the representative energy harvesting devices [20] using the photoelectric effect theory. However, the solar cells have drawbacks such as high weather-dependence and geographical limitations. New energy harvesting devices are required to alternate the solar cells, giving rise to interest in mechanical energy harvesting.

Two approaches for the mechanical energy harvesting, which can be generated by various human movements [21] and wind [22], and piezoelectric [23] and triboelectric [24] effects. Piezoelectric nanogenerators (PENGs) generate electrical signals upon receiving external stress or vibration [9, 25–28]. Triboelectric nanogenerators (TENGs) use an electrostatic phenomenon generated by the contact of two different materials, leading to a potential difference between the materials [29–31]. Various triboelectric nanogenerators have been reported using human mechanical stimuli and various natural energies, such as wind, ultrasonic, raindrops, and water waves [22]. Wind has recently been utilized as a mechanical energy source to flutter the dielectric film for wind-driven TENGs because wind is clean, abundant, ubiquitous, and sustainable [32–34]. The wind-driven TENGs have advantages of simple manufacturing at low cost, low weight, and no explosion risk compared to the lithium-ion batteries [35–38].

Interest in mobility is growing in modern era; for example, drones with numerous wireless sensors are used in a wide variety of fields, including the observation of activities, video filming, and offline delivery [39–41]. Accordingly, an auxiliary power supply providing sustainable energy is required to increase the limited flight time of drones, which are indispensable in the fourth industrial age [42].

Herein, we propose a wind-driven TENG structure with a cross-shaped dielectric film bent in four directions (C-TENG), which can produce a suitable external power supply in all wind directions, unlike the vehicles or subways that can run in only one direction. The TENG could efficiently harvest wind energy from all direction, suitable for drones flying in arbitrary directions. Additionally, another TENG, in which an Al layer is intercalated within a polytetrafluoroethylene (PTFE) film (CIA-TENG), was produced to improve electrostatic induction, resulting in improved triboelectric performances. Al and PTFE were selected as the triboelectric materials. The triboelectric performances of C-TENG and CIA-TENG were compared in this study. The effect of wind direction on the triboelectric performances was also tested, demonstrating that the wind energy can be harvested omnidirectionally.

## Experimental details

### C-film and CIA-film fabrication

A PTFE film tape (3 M™, thickness of 50 μm) was used as a triboelectric material. The C-film was produced by sticking two PTFE film tapes each other and cutting them in the form of a cross-shape. The central square area of the C-film is 2 × 2 cm^2^. An Al foil (HANSUNG, thickness of 15 μm) was used for the intercalated metal layer within the PTFE film. The CIA-film was produced by attaching the two PTFE tapes to both sides of the Al foil. The Al foil was protruded 5 mm from the PTFE film for the connection to the Al bottom electrode electrically. Dry etching was performed using a reactive ion echer (Plasmart, MINIPLASMA station) under following conditions; O_2_, CF_4_, and Ar gases (10, 30, and 15 sccm, respectively), 20 mTorr, and 400 W for 60 s.

### CIA-TENG fabrication

The Al tape (DUCSUNG HITECH, thickness of 50 μm) was used both top and bottom electrodes as well. After bending the cross-shaped CIA-film in four directions, the Al foil protruded from the CIA-film was electrically connected to the Al tape (attched to the bottom glass plate working as the bottom electrode) with a conductive carbon adhesive tape (SHILPA ENTERPRISES, thickness of 15 μm). Another Al tape (for the role of top electrode) was attached to the top glass plate with a size of 2 × 2 cm^2^.

### Triboelectric performance measurements

An oscilloscope (Tektronix, DPD4014B) and pre-amplifier (Stanford Research, SR570) were used to measure the output voltage and current. During the measurement, wind was supplied with a commercial nitrogen blow gun, and the wind speed was measured using a commercial anemometer (Testo, Testo 417). The PTFE surface roughness was analyzed using AFM equipment (Park’s system, XE-100).

## Results and discussion

### Structure design and triboelectric materials for C-TENG and CIA-TENG

**Fig. 1:**
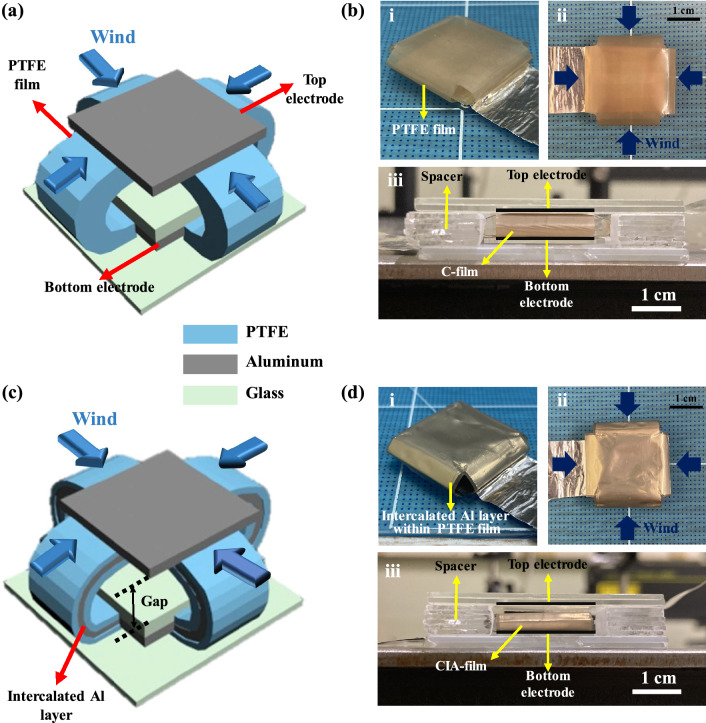
Illustrations and photographs of the fabricated **a**, **b** C-TENG and C-film, and **c**, **d** CIA-TENG and CIA-film; (i) diagonal and (ii) top view of C- and CIA-film, and (iii) side view of the C- and CIA-TENG

Figure 1 shows the structures and photographs of two wind-driven TENGs using different dielectric films bent into four directions for pursuing the omnidirectional fluctuation by the wind. PTFE and Al were used as the triboelectric materials; the PTFE is a dielectric polymer composed of monomers of tetrafluoroethylene, which has abundant fluorines (F). The F atom has the highest electron affinity among many atoms, resulting in accumulated electron charges on the PTFE surface after touching the Al electrode, which is positively charged by electrification. The Al acts as bottom- and top electrodes as well in this study; thus, it simplifies the manufacturing process of the TENG. Inductively coupled plasma-reactive ion etching (ICP-RIE) was performed on the PTFE surface to increase the contact surface area for enhancing the triboelectric performance. The RMS roughness of the PTFE surface was measured as 35.1 nm (before etching) and 53.0 nm (after etching) at a scan area of 15 × 15 µm^2^ (Additional file 1: Fig. S1).

The C-TENG with the cross-shaped PTFE film (C-film) bent in four directions is depicted and shown in Fig. 1a and b. The contact friction is generated by the wind-driven fluctuation of the C-film at the interface with the Al top electrode. The cross-shaped C-film allows the TENG to collect mechanical energy regardless of wind direction and is suitable for vehicles (e.g., drones) moving in arbitrary directions. The effective triboelectric contact area of the C-film is the center part (2 × 2 cm^2^) that actually touches the Al top electrode. Figure 1c and d show another TENG with the intercalated Al layer within the C-film (CIA-film) that is hereafter called CIA-TENG. These two device structures are identical except for the existence of the intercalated Al layer within the PTFE dielectric film. The four wings of CIA-film were bent in four directions and electrically connected to the Al bottom electrode through the protruded intercalated Al layer. The fixed CIA-film is not free from lateral movement but has an elasticity advantageous for the up/down vertical vibration by the wind (Additional file 2: Movie S1).

### Triboelectric performance of C-TENG and CIA-TENG

**Fig. 2:**
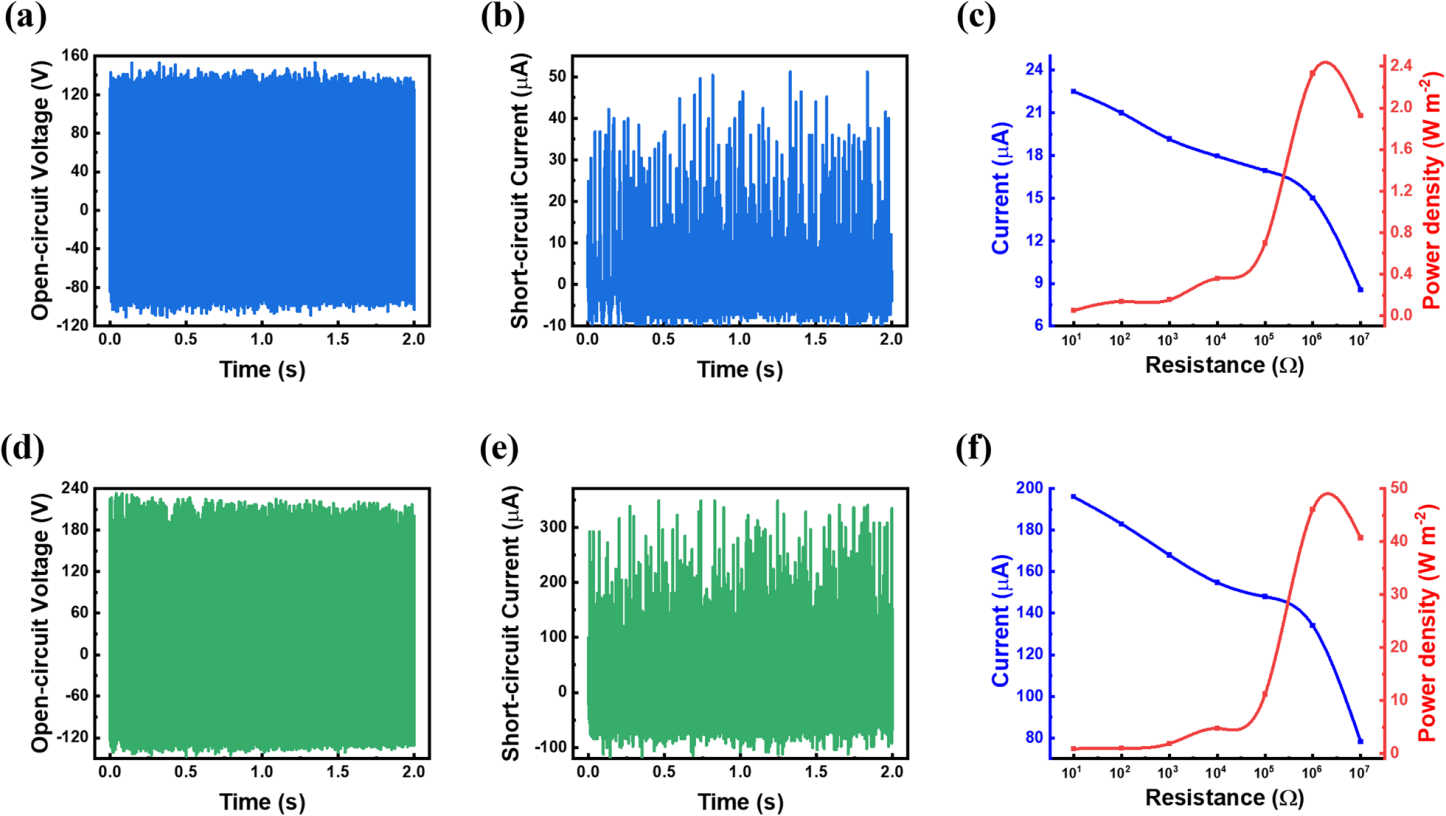
Output performances of the C-TENG and CIA-TENG using the etched PTFE film at a wind speed of 9 m s^− 1^; V_oc_, I_sc_ and output power density of **a**–**c** C-TENG, and **d**–**f** CIA-TENG, respectively

Figure 2 shows the triboelectric performances of the etched C-TENG and CIA-TENG at a wind speed of 9 m s^-1^. In the case of the C-TENG, a maximum open-circuit voltage (V_oc_) of 153 V and short-circuit current (I_sc_) of 51.8 µA were measured (Fig. 2a and b). For the CIA-TENG, the maximum V_oc_ and I_sc_ were 233 V and 348 µA, respectively, which were 1.52 and 6.72 fold larger than those of the C-TENG (Fig. 2d and e). External resistances to the TENGs were applied to measure the output power density. At an external resistance of 1 MΩ, the maximum output power density of the CIA-TENG was 46.1 W m^-2^, which is approximately 20-fold higher than the 2.33 W m^-2^ of the C-TENG (Fig. 2c and f). The I_sc_ increased noticeably when using the intercalated Al layer. A maximum average output power derived from the root-mean-square (RMS) voltage was 2.36 mW at an external resistance of 1 MΩ in CIA-TENG (Additional file 1: Fig. S2).

To identify the effect of surface roughness, two TENGs with the unetched C-film and CIA-film were also measured. The maximum V_oc_ and I_sc_ of the unetched C-TENG were 104 V and 24.4 µA, respectively. In the case of unetched CIA-TENG, those were 149 V and 126.4 µA, respectively (Additional file 1: Fig. S3). These triboelectric properties are significantly inferior to those of corresponding references with the etched dielectric film (Fig. 2). The improved PTFE surface roughness increases the contact surface area to the Al top electrode, resulting in more triboelectric charges on the PTFE surface. It is noticeable that the degree of short-circuit current enhancement is larger than that of open-circuit voltage enhancement, which can be explained by the following equation: [43]

1 $$V=-\frac{Q}{{S}_{eff }{\epsilon }_{0 }}\left({d}_{0}+x\left(t\right)\right)+\frac{{\sigma }_{tribo} x\left(t\right)}{{\epsilon }_{0 }}$$

where, *S*_*eff*_, *d*_*0*_, *σ*_*tribo*_, and *x(t)* are the effective contact area of the dielectric film to the electrode, the effective dielectric thickness constant (d/ε, d: dielectric thickness, ε: relative dielectric constant), triboelectric charges, and the separation distance depending on the time, respectively. In the open-circuit condition, the transferred charges (Q) between electrodes are 0; therefore, the V_oc_ is affected by only *σ*_*tribo*_ and thus linearly proportional to only *σ*_*tribo*_ by Eq. (1). At a short-circuit condition, I_sc_ is given by: [43]

2 $${I}_{sc}=\frac{d{Q}_{sc}}{dt}=-\frac{{S}_{eff}{\sigma }_{tribo}{d}_{0}}{{\left({d}_{0}+x\left(t\right)\right)}^{2}}\frac{dx}{dt}=\frac{{S}_{eff}{\sigma }_{tribo}{d}_{0} v\left(t\right)}{{\left({d}_{0}+x\left(t\right)\right)}^{2}}$$

where *v(t)* is the fluttering speed of the dielectric film. According to Eq. (2), I_sc_ is linearly proportional to the multiplied value of *S*_*eff*_ and *σ*_*tribo*_. The etching process increased the surface roughness, inducing more contact surface area during electrification, and more triboelectric charges on the PTFE surface, resulting in the higher increment of I_sc_ in comparison to the V_oc_ increment.

### Operating mechanism of the C-TENG and CIA-TENG

**Fig. 3:**
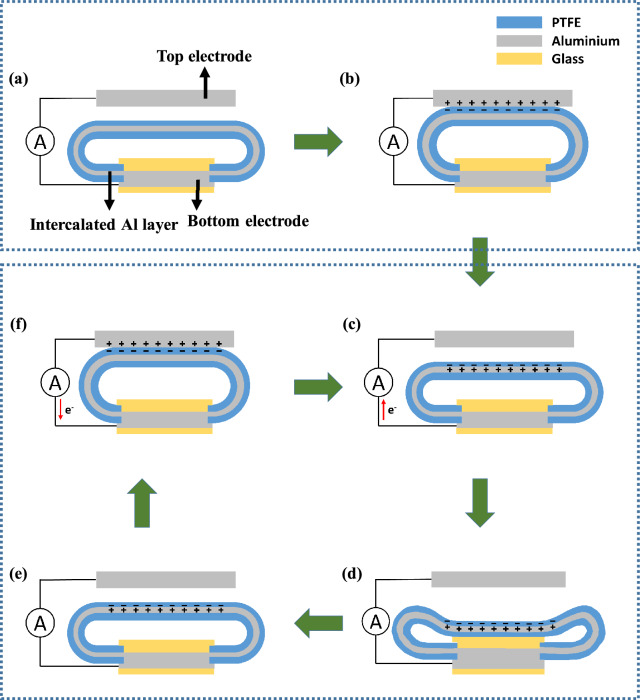
Operating mechanisms of CIA-TENG; **a**, **b** initial electrification step and **c**–**f** repetitive triboelectric step

To understand the effect of the intercalated Al layer, the operating mechanism of the C-TENG and CIA-TENG was analyzed as depicted in Additional file 1: Fig. S4 and Fig. 3. Two primary mechanisms are used for the operation of the wind-driven TENG. The first mechanism is the electrification of the dielectric film through the contact between the two triboelectric materials. The second step is electrostatic induction, in which the free electrons at the electrode surface are repulsed by the negative triboelectric charges sitting on the dielectric film when it approaches the electrode. As a result, an equal amount of positive charges remains on the electrode surface.

In the C-TENG, when the C-film contacts the Al top electrode, negative triboelectric charges are accumulated on the surface of the C-film by withdrawing free electrons from the Al top electrode (Additional file 1: Fig. S4a and b). During the initial electrification cycles, triboelectric charges are accumulated on both surfaces of the C-film and Al top electrode. When detaching, as the distance between the C-film and Al top electrode increases, the electrostatic induction to the Al top electrode by the negative charges of the C-film is gradually weakened, resulting in a flow of electrons from the bottom to the top electrode (Additional file 1: Fig. S4c) until the electrostatic induction is no longer active on the surface of the Al top electrode (Additional file 1: Fig. S4d). On the contrary, when the negatively charged C-film approaches the Al top electrode, electrons move from the top to the bottom electrode, because Coulomb repulsion occurs between the negative triboelectric charges of the C-film and the free electrons of the Al top electrode (Additional file 1: Fig. S4e and f).

The same mechanism is applicable to the CIA-TENG during the initial few cycles, giving rise to negative triboelectric charges on the CIA-film surface (Fig. 3a and b). As soon as the negatively charged CIA-film is detached from the Al top electrode, more electrons move from the bottom to the top electrode than those of the C-TENG because sudden electrostatic equilibrium was generated at the interface between the negatively charged CIA-film and the intercalated Al layer, generating positive charges on the intercalated Al surface (Fig. 3c). Concurrently, the Coulomb repulsion between the triboelectric electrons of the CIA-film and free electrons of the intercalated Al layer occurs. These two combined effects (electrostatic induction and Coulomb repulsion) induce more electrons’ flow to the Al top electrode until electrostatic equilibrium is reached at which the net current is zero (Fig. 3d and e). Therefore, the intercalated Al layer within the PTFE film generates more electricity in comparison to the C-TENG. When the CIA-film touches the top electrode in the next oscillation, electrons immediately move from the top electrode to the intercalated Al layer to maintain electrostatic equilibrium at the interface between the CIA-film and Al top electrode (Fig. 3f). This triboelectric mechanism continues in the following oscillation. A detailed explanation of the effect of intercalated Al layer within the PTFE film was given in a previously reported paper [44].

### Effect of gap, wind speed, and wind direction on the triboelectric performances of the CIA-TENG

**Fig. 4:**
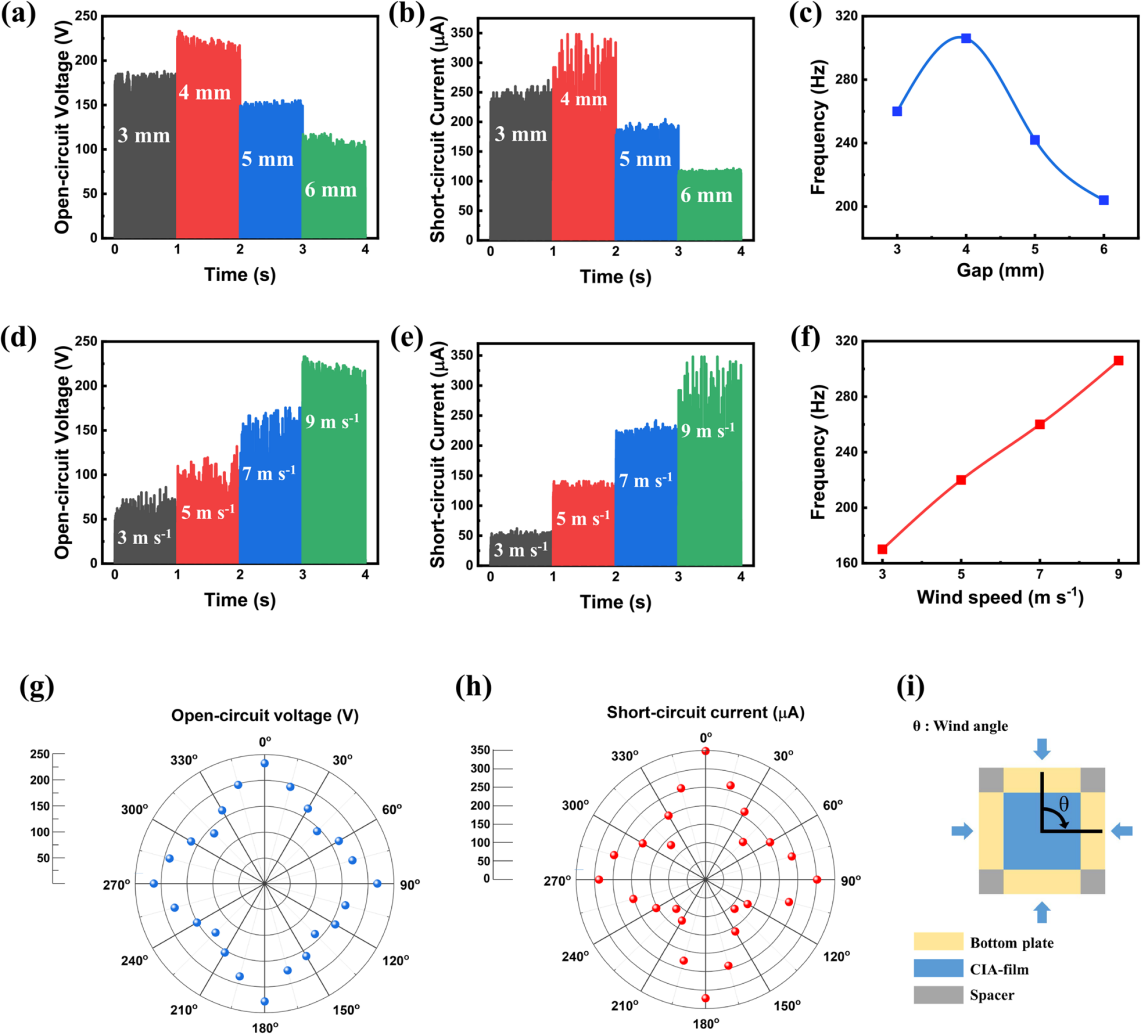
Triboelectric performances of the CIA-TENG at various **a**–**c** gaps, **d**–**f** wind speeds, and **g**–**i** wind directions shown from the top view of CIA-TENG

The CIA-TENG was operated under various conditions to check the effect of the gap, wind speed, and wind direction. Figure 4a and b show the V_oc_ and I_sc_ of CIA-TENG measured at various gaps. The V_oc_ and I_sc_ were 188, 233, 155, and 120 V and 271, 348, 205, and 122 µA at the gap of 3, 4, 5, and 6 mm, respectively, revealing the maximum triboelectric performance at a gap of 4 mm. The fluttering frequencies of CIA-film were 260, 306, 242, and 204 Hz at the corresponding gaps of 3, 4, 5, and 6 mm at a wind speed of 9 m s^-1^ (Fig. 4c). The fluttering frequency was calculated by counting the number of I_sc_ peaks at an interval of 0.05 s (Additional file 1: Fig. S5). The highest fluttering frequency of 306 Hz was measured at a gap of 4 mm at which the best wind-driven triboelectric performance was demonstrated. The narrower gap (3 mm) disturbed the fluttering of the CIA-film, resulting in a lower fluttering frequency, which implies that the dielectric film impacts the Al top electrode with a lower speed of *v(t)*, rendering a drop in I_sc_ according to Eq. (2). The V_oc_, I_sc_, and fluttering frequency were measured at various wind speeds (Fig. 4d–f). As expected, the fluttering frequency increased with the wind speed; thus, both V_oc_ and I_sc_ increased with wind speed.

To verify that the CIA-TENG enables energy harvesting in all wind directions, the triboelectric performance was measured depending on the wind angles (Fig. 4 g and h). The wind angle (θ) was defined as illustrated in Fig. 4i. The maximum V_oc_ and I_sc_ were revealed at 0, 90, 180, and 270°, where the wind blows vertically to the CIA-film plane. In contrast, the minimum V_oc_ and I_sc_, which were approximately 58 and 32 % of the maximum V_oc_ and I_sc,_ respectively, were recorded at 45, 135, 225, and 305°, where the wind blows diagonally to the void between the CIA-films. These results indicate that wind energy can be harvested regardless of the wind direction with the cross-shaped dielectric film.

### Application of the CIA-TENG

**Fig. 5:**
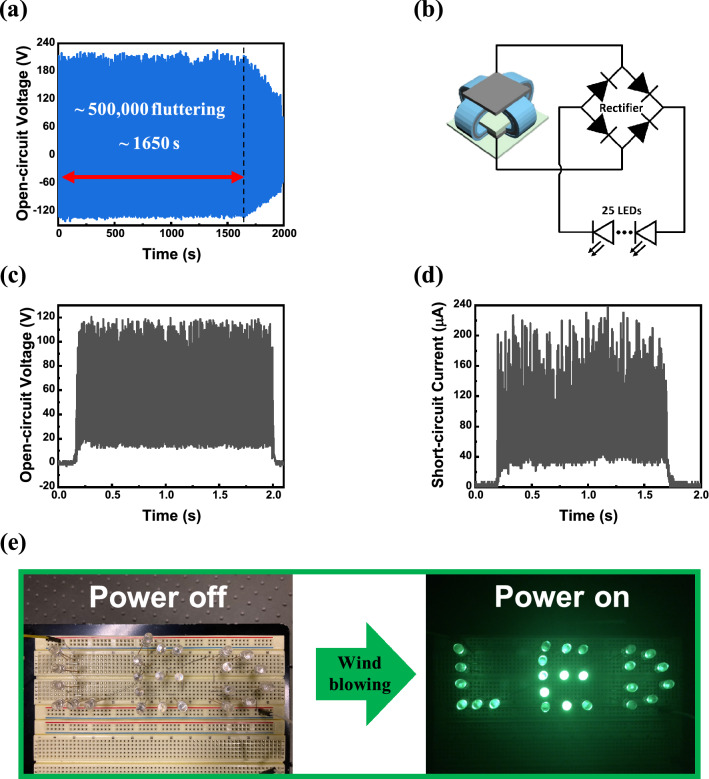
**a** The change of V_oc_ of the CIA-TENG at a wind speed of 9 m s^− 1^ for 500,000 fluttering cycles. **b** A schematic circuit diagram for powering 25 LEDs and **c**, **d** the triboelectric performances of the CIA-TENG with a bridge rectifier in series. **e** Snapshot of the lit “LED” characters composed of 25 LEDs, powering by the CIA-TENG

A continuous operation was performed at a wind speed of 9 m s^-1^ to test the durability of the CIA-TENG. Figure 5a shows that the V_oc_ was maintained for approximately 1650 s, corresponding to 500,000 fluttering cycles, and gradually decreased. For practical application, the CIA-TENG was connected to a bridge rectifier to convert the AC to a DC signal, and 25 light-emitting diodes (LED) bulbs in series (Fig. 5b). The resulting maximum V_oc_ of 120.4 V and I_sc_ of 237.6 µA were 48 and 32 % lower than those of the AC signal, respectively (Fig. 5c and d). The CIA-TENG supplied a stable power to 25 LEDs to light “LED” characters (Fig. 5e and Additional file 3: Movie S2).

## Conclusions

In this study, wind-driven C-TENG and CIA-TENG were fabricated and their triboelectric performances were compared. Cross-shaped dielectric films bent in four directions were proposed to harvest the wind energy regardless of wind directions. By inserting an Al layer within the dielectric film, the combined effects of electrostatic equilibrium and Coulomb repulsion between the CIA-film and the intercalated Al layer generated more electrons for the triboelectric performance. When comparing the triboelectric performances of the C-TENG and CIA-TENG, the V_oc_, I_sc_, and output power density of the CIA-TENG were 1.52, 6.72, and 20 times higher than those of the C-TENG, respectively. The CIA-TENG could harvest energies sustainably by the wind blowing in arbitrary directions; the CIA-TENG could be installed and supply an auxiliary power to the vehicles moving in arbitrary ways.

## Supplementary Information

**Additional file 1**:** Fig. S1**. AFM images of PTFE films (a) before and (b) after RIE etching.** Fig. S2**. (a) Maximum and RMS output voltage, and (b) average power vs. external resistance.** Fig. S3**. Voc and Isc of (a,b) the C-TENG and (c,d) the CIA-TENG using the unetched PTFE film at a wind speed of 9 m s^-1^.** Fig. S4**. Operating mechanisms of C-TENG; (a,b) initial electrification step and (c–f) repetitive triboelectric step.** Fig. S5**. Comparison of Isc at different (a) gaps and (b) wind speeds (interval: 0.05 s.

**Additional file 2**:** Movie S1**. Vertical vibration of the CIA-TENG at a wind speed of 9 m s^-1^.

**Additional file 3**:** Movie S2**. Demonstration of powering 25 LEDs by a CIA-TENG.

## Data Availability

The datasets used and/or analyzed during the current study are available from the corresponding author on reasonable request.

## Abbreviations

TENG: Triboelectric nanogenerator
Al: Aluminum
V_oc_: Open-circuit voltage
I_sc_: Short-circuit current
IoT: Internet of Thing
C-TENG: Wind-driven TENG structure with a cross-shaped dielectric film bent in four directions
PTFE: Polytetrafluoroethylene
CIA-TENG: C-TENG in which an Al layer is intercalated within a PTFE film
F: Fluorine
ICP-RIE: Inductively coupled plasma-reactive ion etching
C-film: Cross-shaped PTFE film
CIA-film: C-film having the intercalated Al layer
RMS: Root-mean-square
V_rms_: RMS voltage
P_avg_: Average output power
LED: Light-emitting diode
