# Supplementary material for: Omni-directional wind-driven triboelectric nanogenerator with cross-shaped dielectric film
Source: Nano Converg. 2021 Sep 2;8:25. doi: 10.1186/s40580-021-00276-5 (PMC8413407; doi:10.1186/s40580-021-00276-5)
Supplement: Supplementary file 1 — Additional file 1: Fig. S1. AFM images of PTFE films (a) before and (b) after RIE etching. Fig. S2. (a) Maximum and RMS output voltage, and (b) average power vs. external resistance. Fig. S3. Voc and Isc of (a,b) the C-TENG and (c,d) the CIA-TENG using the unetched PTFE film at a wind speed of 9 m s-1. Fig. S4. Operating mechanisms of C-TENG; (a,b) initial electrification step and (c–f) repetitive triboelectric step. Fig. S5. Comparison of Isc at different (a) gaps and (b) wind speeds (interval: 0.05 s. [file 40580_2021_276_MOESM1_ESM.docx]

**Supplementary Information**

**Omni-directional wind-driven triboelectric nanogenerator with cross-shaped dielectric film**

Yoseop Shin^a,1^, Sungjun Cho^a,1^, Sejin Han^a^, Gun Young Jung^a,^*

^a^School of Materials Science and Engineering, Gwangju Institute of Science and Technology (GIST), 123 Cheomdan-gwagiro, Buk-gu, Gwangju, 61005, Republic of Korea

^1^These authors equally contributed.

*Corresponding author

Tel.: +82-62-715-2324

E-mail address: [gyjung@gist.ac.kr](mailto:gyjung@gist.ac.kr)

**Fig. S1.** AFM images of PTFE films (a) before and (b) after RIE etching.

**Fig. S2.** (a) Maximum and RMS output voltage, and (b) average power vs. external resistance.

The maximum average power is calculated by RMS voltage (V_rms_) at an optimal external load. The V_rms_ at the external load is calculated by using the following equation:

$V_{rms}=\sqrt{\frac{1}{T}\int{V(t)}^{2}dt}$ (1)

where, *T* is the total measurement time and *V(t)* is the measured voltage over time. Fig. S2 shows the triboelectric outputs at various external resistances. As shown in Fig. S2a, both the maximum and RMS voltage tended to increase with the external resistance. The average output power was calculated by using the following equation:

$P_{avg}=\frac{V_{rms}^{2}}{R}$ (2)

where, P_avg_ is average output power and R is total external resistance including the input impedance (50 Ω) of oscilloscope. As a result, a maximum P_avg_ of 2.36 mW was achieved at an external resistance of 1 MΩ (Fig. S2b). The input impedance (50 Ω) of oscilloscope is much lower than the optimal external resistance, making a negligible effect on the P_avg_.

**Fig. S3.** V_oc_ and I_sc_ of (a,b) the C-TENG and (c,d) the CIA-TENG using the unetched PTFE film at a wind speed of 9 m s^-1^.

**Fig. S4.** Operating mechanisms of C-TENG; (a,b) initial electrification step and (c–f) repetitive triboelectric step.

**Fig. S5.** Comparison of I_sc_ at different (a) gaps and (b) wind speeds (interval: 0.05 s

**Movie S1.** Vertical vibration of the CIA-TENG at a wind speed of 9 m s^-1^.

**Movie S2.** Demonstration of powering 25 LEDs by a CIA-TENG.
